# Sphingosine Kinase Blockade Leads to Increased Natural Killer T Cell Responses to Mantle Cell Lymphoma

**DOI:** 10.3390/cells9041030

**Published:** 2020-04-21

**Authors:** Michael S. Lee, Wenji Sun, Tonya J. Webb

**Affiliations:** 1 Department of Microbiology and Immunology, University of Maryland School of Medicine, Baltimore, MD 21201, USA; 2 Marlene and Stewart Greenebaum Comprehensive Cancer Center, University of Maryland School of Medicine, Baltimore, MD 21201, USA

**Keywords:** Mantle cell lymphoma, NKT cells, sphingosine kinase, sphingosine-1-phosphate, cardiolipin

## Abstract

Mantle cell lymphoma (MCL) is an aggressive subtype of non-Hodgkin’s lymphoma. Despite being responsive to combination chemotherapy, median survival remains around 5 years due to high rates of relapse. Sphingolipid metabolism regulates MCL survival and proliferation and we found that sphingosine-1-phosphate (S1P) is upregulated in MCL cells. Therapeutic targeting of the S1P_1_ receptor or knockdown of sphingosine kinase 1 (SK1), the enzyme responsible for generating S1P, in human MCL cells results in a significant increase in Natural Killer T (NKT) cell activation. NKT cells recognize glycolipid antigens presented on CD1d and can reduce MCL tumor burden in vivo. Lipidomic studies identified cardiolipin, which has been reported to bind to CD1d molecules, as being upregulated in SK1 knockdown cells. We found that the pretreatment of antigen presenting cells with cardiolipin leads to increased cytokine production by NKT cell hybridomas. Furthermore, the ability of cardiolipin to activate NKT cells was dependent on the structure of its acyl chains. Collectively, these studies delineate novel pathways important for immune recognition of malignant cells and could lead to the development of new treatments for lymphoma.

## 1. Introduction

Mantle cell lymphoma (MCL) is characterized by its genetic hallmark, the chromosomal translocation t(11;14), which results in increased expression of cyclin D1 in B cells, alterations in the DNA damage response, and constitutive activation of key anti-apoptotic pathways, such as phosphatidyl-inositol 3-kinase (PI3K)/Akt and nuclear factor-kB (NF-kB) [1]. Collectively, these changes result in cell cycle dysregulation and give rise to profound genetic instability [2]. Despite being initially responsive to combination chemotherapy, relapsed patients have an overall survival of less than three years [3]. Therefore, it is essential to explore new treatment options targeting the numerous dysregulated pathways that are operable in MCL. Immunomodulatory therapy directed towards increasing immunologic killing of MCL cells, as well as boosting the numbers of cancer-directed cytotoxic cells offers the possibility of long-term responses.

Natural killer T cells (NKT cells) are a subset of innate-like T cells that express certain natural killer cell receptors and serve as a link between the innate and adaptive immune systems. NKT cells are early producers of large amounts of Th1, Th2, and Th17 cytokines, and can directly mediate cytotoxicity [4,5,6]. NKT cells have been demonstrated to play a role in autoimmune disease [7], tumor surveillance [6,8], hematological cancers [9], infectious disease, and inflammatory conditions such as ischemia reperfusion injury [10]. NKT cells are characterized by a semi-invariant T cell receptor (TCR) (Vα14Jα18 in mice and Vα24Jα18 in humans). Unlike conventional CD4 and CD8 T cells, NKT cells are activated by glycolipid antigens presented in the context of the non-classical, class I major histocompatibility complex (MHC)-like molecule, CD1d [11,12]. α-Galactosylceramide (α-GalCer) is a potent activator of NKT cells [13,14], and following treatment with α-GalCer, NKT cells produce cytokines, undergo clonal expansion, and subsequently activate other immune cells [15,16,17,18,19]. After activation, human NKT cells can exhibit strong anti-tumor activity against many tumors [20,21]. Several groups have conducted clinical trials evaluating the effectiveness of α-GalCer as a potential therapeutic immunomodulator of NKT cells that yielded modest results, potentially due to a reduction in NKT cell number and function in cancer patients [22,23,24,25,26,27].

Sphingosine-1-phosphate (S1P) regulates proliferation, survival, and migration of mammalian cells through both extracellular receptor-mediated and intracellular mechanisms [28,29,30]. S1P is generated from sphingolipids, essential serum membrane lipids which are concentrated in lipid rafts. There are several enzymes in this pathway, which culminates in sphingosine kinase (SK) conversion of sphingosine into S1P. S1P is involved in malignant transformation, cancer cell proliferation, inflammation, vasculogenesis, lymphocyte trafficking, and resistance to apoptosis. There are two forms of sphingosine kinase: SK1 and SK2. Over-expression of SK1 has been reported to induce malignant transformation and tumor formation in 3T3 fibroblasts [31], while SK2 has been shown to promote acute lymphoblastic leukemia by increasing MYC expression [32]. In addition, SK1 has been shown to be associated with tumor growth and poor outcomes in humans [33,34,35,36,37,38]. It is known that S1P has a major regulatory role in supporting the circulating lymphocyte population [39]. Results from one study implied a critical role for S1P signaling in MCL pathogenesis, as inhibition of S1P signaling via treatment with FTY-720 (an immunosuppressant that binds to four known S1P receptors (S1P_1_, _3-5_) and inhibits downstream signaling) resulted in the time- and dose-dependent cytotoxicity of MCL tumor cells [40]. However, the effect S1P plays in anti-tumor immune responses to MCL has yet to be determined. 

Here, we present evidence demonstrating that S1P signaling alters CD1d-dependent antigen processing and presentation resulting in diminished NKT cell responses. We also show that blocking S1P signaling or knockdown of SK leads to NKT cell-mediated killing of MCL. Knockdown of SK alters the lipid repertoire of MCL, and we have identified a unique cardiolipin species that is increased by SK knockdown and acts as an activating lipid for NKT cells. Our data suggests that SK activity and subsequent S1P signaling by B cell lymphomas serves to facilitate evasion of lymphoma detection by NKT cells. To our knowledge this is the first report demonstrating that SK activity and S1P signaling by lymphomas can suppress CD1d-mediated NKT cell responses.

## 2. Materials and Methods

### 2.1. Cell Lines

DN32.D3 and N38-3C3 are type 1 NKT cell hybridoma cell lines [41]. The cells were cultured in the presence of IMDM supplemented with 5% FBS, l-glutamine, and penicillin-streptomycin (100 units/mL—100 µg/mL). L-CD1d cells were derived from the LMTK mouse fibroblast cell line and were transfected to express high levels of mouse CD1d. These cells were cultured in DMEM with 10% FBS, 2 mM l-glutamine, penicillin-streptomycin (100 units/mL—100 µg/mL), and G418 sulfate (500 μg/mL). LMTK cells transfected with an empty vector were used as controls [42]. The MCL lines (SP53 and Jeko-1) were kindly provided by Dr. Raymond Lai (University of Alberta, Edmonton, Canada). C1R-CD1d, a lymphoma cell line stably transfected with human CD1d cDNA was graciously provided by Dr. Mark Exley (Harvard Medical School, Boston, MA). Healthy donor NKT cell lines were kindly provided by Dr. Moriya Tsuji (The Rockefeller University, New York, NY) [10]. Phenotypic characterization of all cell lines was confirmed by flow cytometry.

### 2.2. Human Samples

Healthy donor sera samples (n = 10) were purchased from Innovative Research (Novi, MI, USA). MCL patient plasma samples (n = 14) were collected from patients undergoing treatment at the Marlene and Stewart Greenebaum Cancer Center at the UMSOM. The clinical diagnosis of MCL was confirmed in our patient population using cytogenetics. All donors gave written informed consent before enrolling in the study. The Institutional Review Board at the University of Maryland School of Medicine (UMSOM) approved this investigation.

### 2.3. Primary Mouse Cells

All animal studies were conducted with the approval of the University of Maryland Institutional Animal Care and Use Committee (IACUC). Spleens and livers were isolated from C57BL/6J mice (bred in house) and pushed through a 70-µm cell strainer to generate single cell suspensions. Liver mononuclear cells (MNCs) were separated from hepatocytes using Percoll (GE Healthcare, 17-0891-01), as previously described [43]. The cells were then incubated in ACK Lysing Buffer (Quality Biological, 118-156-721) to remove red blood cells, washed, and counted prior to co-culture setup.

### 2.4. S1P Modulators, Lipids, and Reagents

SEW2871 and W146 were purchased from Cayman Chemicals. The cardiolipin mixture isolated from bovine heart pericardium were obtained from Sigma (C1649-10MG). 18:1 cardiolipin and 16:1 cardiolipin were obtained from Avanti (710335 and 710339). S1P ELISAs were conducted in our laboratory and by Dr. Parsons’ laboratory at the LSU School of Medicine using the S1P ELISA Kit (Echelon, K-1900) according to the manufacturer’s instructions.

### 2.5. Cell Growth Assays

MCL cell lines were plated at 1e5 cells/well in 100 µL of media containing 1:10 diluted WST-1 reagent (Takara Bio, MK400). Absorbance at 440 nm was measured periodically and graphed. Each condition was conducted in triplicate.

### 2.6. Co-Culture Studies

For S1P and cardiolipin, antigen presenting cells (APCs) were pulsed for the indicated time period at 37 °C. The APCs were then washed with ice cold 1x PBS and set up in co-culture with NKT cell hybridomas overnight. Then the cells were spun down and the level of IL-2 in the supernatant was determined by ELISA (BD Biosciences, 555148). For the S1P receptor studies, the MCL cell lines Jeko-1 and SP53 were incubated with 10 µM SEW2871 (S1P_1_ agonist) or the S1P_1_ antagonist, W146, for 72 h. The cells were then washed and set up in co-culture with primary NKT cells. After co-culture, IFN-γ levels in the supernatant were determined by ELISA (BioLegend, 430101).

### 2.7. Cytotoxicity Assays

MCL cell lines were incubated with primary human NKT cells at the indicated ratios in the presence of antigen, α-GalCer (100 ng/mL) (Avanti, 867000) for 20–24 h. NKT cell mediated cell lysis was assessed by standard ^51^Cr-release assay.

### 2.8. RT-PCR

RNA was extracted using the RNeasy kit (Qiagen, Hilden, Germany). cDNA was reverse transcribed by using iScript select cDNA Synthesis Kit (BioRad, Hercules, CA, USA). PCR was performed as described [44]. In brief, mRNA was detected by RT-PCR with primers listed as follows: hβ-actin, forward, 5′-ATCTGGCACCACACCTTCTACAATGAGCTGCG-3′, reverse, 5′-CGTCATACTCCTGCTTGCTGATCCACATCTCG-3′, S1P_1_, forward, 5′-AGCGTTCGTCTGGAGTAG-3′; reverse, 5′-TCAATGGCGATGGCGAGGAG-3′; S1P_4_, forward, 5′-GAACATCACGCTGAGTGAC-3′, reverse, 5′-AGATCACCAGGCAGAAGAG-3′. The thermal cycling conditions comprised of an initial denaturation step at 95 °C for 10 min followed by 35 cycles of PCR using the following profile: 94 °C for 30 s, 60 °C for 1 min, and 72 °C for 2 min.

### 2.9. Real Time Quantitative PCR (qPCR) Analysis

Total RNA was isolated from lymphoma cell lines using RNeasy kit (Qiagen) and reverse transcribed by iScript select cDNA Synthesis Kit (BioRad). Real-time quantitative PCR was performed with an ABI prism 7300 Thermal Cycler (Applied Biosystems) using TaqMan Gene Expression Master Mix and TaqMan Gene expression assays (Product No. 4331182 both from Applied Biosystems) to amplify human SphK1 (Hs00184211_m1) and human SphK2 (Hs00219999_m1) according to the manufacturer’s instructions. Data was quantitated using ImageJ (NIH) densitometry analysis.

### 2.10. Lipidomic Analysis

Lipidomic analysis was conducted by Lipomics, a division of Metabolon, Inc. Briefly, lipids are extracted from samples in methanol: dichloromethane in the presence of internal standards. The extracts are concentrated under nitrogen and reconstituted in 0.25 mL of 10 mM ammonium acetate dichloromethane: methanol (50:50). The extracts are transferred to inserts and placed in vials for infusion-MS analysis, performed on a Shimazdu LC with nano PEEk tubing and the Sciex SelexIon-5500 QTRAP. The samples are analyzed via both positive and negative mode electrospray. The 5500 QTRAP scan is performed in MRM mode with the total of more than 1100 MRMs. Individual lipid species were quantified by taking the peak area ratios of target compounds and their assigned internal standards, then multiplying by the concentration of internal standard added to the sample. Lipid class concentrations were calculated from the sum of all molecular species within a class, and fatty acid compositions were determined by calculating the proportion of each class comprised of individual fatty acids.

### 2.11. Statistical Analysis

An unpaired two-tailed Student’s *t*-test and one-way ANOVA were performed using Prism software (version 5.02 for Windows; GraphPad) to compare control and experimental groups. A *p*-value < 0.05 was considered significant.

## 3. Results

### 3.1. S1P Pretreatment Inhibits CD1d-Mediated NKT Cell Activation

**Figure 1:**
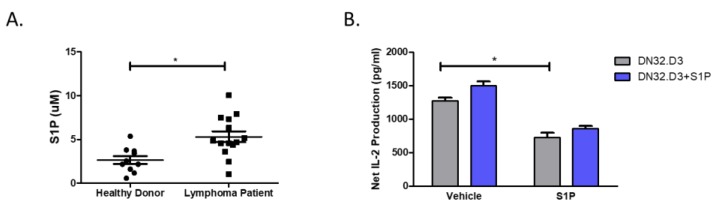
Pretreatment with S1P inhibits CD1d-mediated NKT cell activation. (**A**) S1P levels in healthy donor and MCL patient sera were measured using ELISA. (**B**) NKT cells (DN32.D3) and B cell lymphomas (C1R-CD1d) were pretreated with vehicle (DMSO) or S1P (1 μg/mL) for 1 h at 37 °C. DN32.D3 (5 × 10^4^) NKT cell hybridomas were incubated with C1R-CD1d cells (2.5 × 10^5^) in the presence of α-GalCer (100 ng/mL) for 20–24 h. ELISA was used to measure IL-2 production. Data was analyzed by a two-tailed *t*-test with Welch’s correction. * *p* < 0.05.

We first analyzed healthy donor (n = 10) and MCL patient (n = 14) plasma and found that S1P levels were elevated in MCL patient plasma relative to healthy donors (5.31 ± 0.62 vs. 2.66 ± 0.45 µM; Figure 1A). To examine the effects of S1P on NKT cell activation, C1R-CD1d cells were used as targets and DN32.D3 NKT cell hybridomas served as effector cells. C1R-CD1d cells, DN32.D3, or both cell lines were pre-treated with S1P for an hour. After co-culture, NKT cell activation was determined by IL-2 ELISA. Pretreatment of the NKT hybridomas alone did not alter NKT cell responses compared to untreated cells. However, pre-treatment of our target cells, C1R-CD1d, resulted in a significant decrease in IL-2 production by NKT cells (Figure 1B). The decrease was not altered by additional treatment of the NKT hybridomas. Taken together, these data suggest that S1P inhibits the ability of the target cell to induce NKT cell activation and this pathway may contribute to failure of immune surveillance in MCL.

### 3.2. Targeting of S1P_1_ Signaling Enhances NKT Cell-Mediated Lysis of MCL

**Figure 2:**
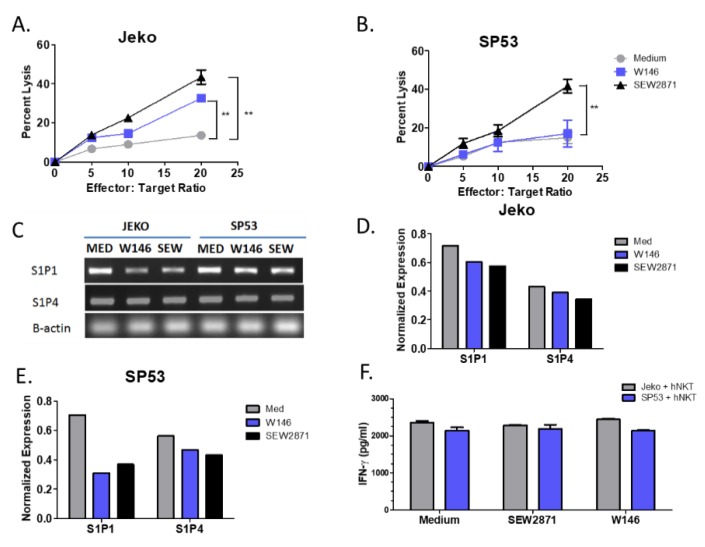
Targeting of S1P_1_ signaling enhances NKT cell-mediated cytotoxicity of MCL. (**A**) Jeko and (**B**) SP53 cells were incubated with 10 µM SEW2871 or W146 for 72 h, washed, and co-cultured with primary NKT cells at the indicated ratios in the presence of α-GalCer (100 ng/mL) for 24 h and NKT cell mediated cell lysis was assessed by standard ^51^Cr-release assay. (**C**) MCL cell lines express S1P receptors. Expression of S1P_1_ and S1P_4_ was determined by RT-PCR after incubation with 10 µM SEW2871 (S1P1 agonist) or the S1P_1_ antagonist, W146, for 72 h. Expression was quantitated using densitometry for S1P1 and S1P4 in (**D**) Jeko and (**E**) SP53 relative to B-actin. (**F**) IFN-γ levels were determined by ELISA. Data are representative of three independent experiments. Data were analyzed by one-way ANOVA. ** *p* < 0.001.

We next examined whether targeting the S1P_1_ receptor on antigen presenting cells directly could alter NKT cell responses. We utilized two different MCL cell lines, Jeko and SP53, as our target cells. Both cell lines expressed the S1P receptor 1 (S1P_1_). Therefore, we investigated the effect of two drugs, SEW2871 and W146, that target S1P_1_ on NKT cell responses to MCL cell lines. Pretreatment of the Jeko MCL cell line with either SEW2871 or W146 increased sensitivity to NKT cell-mediated lysis (Figure 2A). Similarly, pretreatment of the SP53 MCL cell line with SEW2871, but not W146, resulted in increased lysis when co-cultured with human NKT cells (Figure 2B). We next examined the expression of different S1P receptors on each of our MCL cell lines by RT-PCR in the presence or absence of SEW2871 or W146. We found that S1P_1_, to a greater extent than S1P_4_, was downregulated following treatment with either SEW2871 or W146 in both the Jeko and SP53 cell lines (Figure 2C–E). Finally, we found that pretreatment of MCL cells with either SEW2871 or W146 did not alter their ability to induce cytokine production by human NKT cells (Figure 2F). These data demonstrate the therapeutic potential of targeting S1P_1_ due to the enhanced lysis of MCL cell lines by human NKT cells following drug pretreatment.

### 3.3. Knockdown of Sphingosine Kinase Restores NKT Cell Responses to MCL

**Figure 3:**
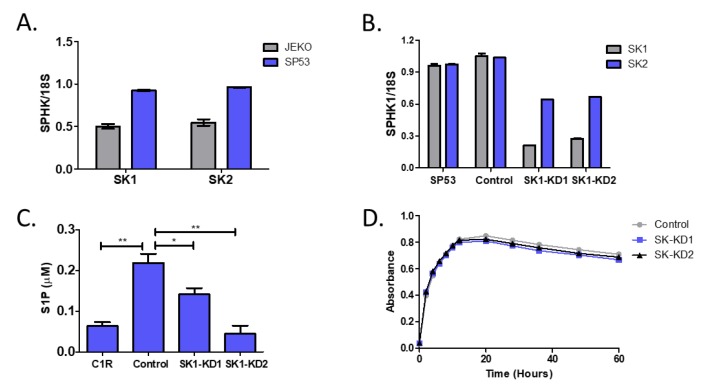
Generation of sphingosine kinase knockdown cell lines. (**A**) mRNA levels of SK1 and SK2 in Jeko and SP53 MCL lines were normalized to 18S expression. (**B**) SP53 cells were stably transfected with shRNA plasmids to knock down expression of sphingosine kinase 1 (SK1) or with a scrambled shRNA control plasmid. Two clones were selected and used for experiments (SK1-KD1 or SK1-KD2). Knockdown of SK was confirmed by quantitative real-time PCR (qPCR). SK1 and SK2 levels were assessed relative to 18S. Expression in the parental cell line (SP53) was compared to the scrambled shRNA control and the SK1-KD clones. (**C**) Reduction of SK results in decreased S1P production. S1P levels in cell lysates were measured by ELISA. (**D**) Control (SP53), SK-KD1, and SK-KD2 cell growth was monitored by WST-1 using absorbance at 440 nm. Error bars are contained within the points. Data was analyzed by a two-tailed *t*-test. * *p* < 0.05; ** *p* < 0.001.

Next, we sought to examine the impact of directly targeting sphingosine kinase (SK). There are two isoforms of SK, SK1 and SK2. We first measured the expression of SK1 and SK2 in the MCL lines Jeko and SP53. There were higher expression levels of both SK1 and SK2 in SP53 cells compared to Jeko cells (Figure 3A). In addition, SP53 cells secreted higher levels of S1P into the cell culture supernatant and had higher intracellular levels of S1P, as measured in cellular lysates by mass spectrometry, compared to Jeko (data not shown). Therefore, we knocked down SK in the SP53 cell line using shRNA. Although we selectively targeted SK1, as shown in Figure 3B, the expression of SK1 and SK2 were reduced in both SP53 clones (SK1-KD1 and SK1-KD2). After confirming that SK was knocked down, we also confirmed that knockdown of SK corresponded to a reduction in S1P production (Figure 3C). In addition, we found that the knock down of SK did not significantly impact cellular growth (Figure 3D).

**Figure 4:**
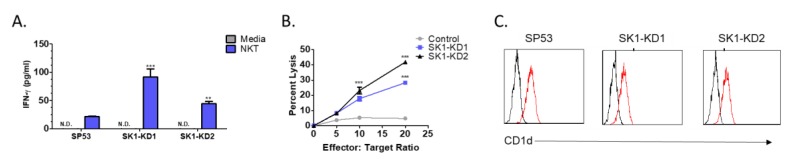
Knockdown of sphingosine kinase restores NKT cell responses to MCL. (**A**) Human NKT cells were co-cultured with SP53 and SK1-KD cells and NKT cell activation, as determined by IFN-γ production, was measured by ELISA. (**B**) NKT cell specific lysis of SP53, SK1-KD1, and SK1-KD2 was assessed by standard ^51^Cr-release assay. (**C**) CD1d expression on SP53, SK1-KD1, and SK1-KD2 cell lines was determined by flow cytometry. Data was analyzed by one-way ANOVA. ** *p* < 0.001; *** *p* < 0.0001.

To determine whether knockdown of SK1 results in enhanced NKT cell responses, SP53-control, SK1-KD1, or SK1-KD2 cells were co-cultured with human NKT cells. We then measured IFN-γ production and cell lysis to determine the level of NKT cell responses. We found that human NKT cells produced more IFN-γ when co-cultured with either SK1-KD1 or SK1-KD2 compared to SP53 (Figure 4A). We also found that human NKT cells were able to lyse both SK1-KD1 and SK1-KD2 to a greater extent than they were able to lyse SP53 (Figure 4B). Finally, we measured the expression of CD1d on SP53, SK1-KD1, and SK1-KD2 by flow cytometry and found all three to have similar expression, confirming that the observed increase in NKT cell responses is not due to changes in surface expression of CD1d (Figure 4C).

### 3.4. Knockdown of Sphingosine Kinase Results in Increased Cardiolipin Levels

**Figure 5:**
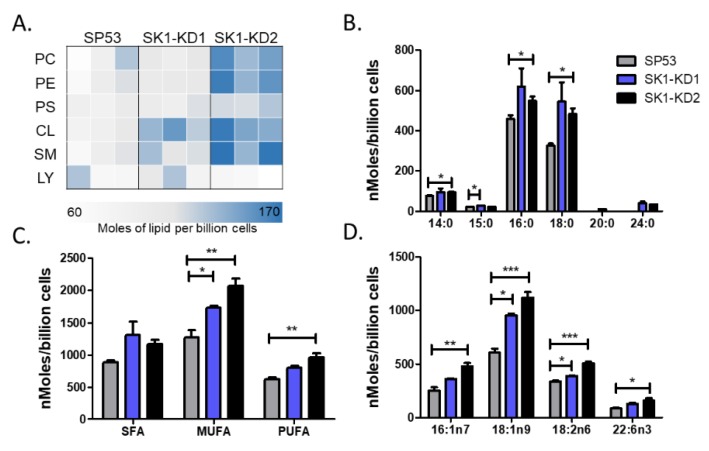
Lipidomic analysis of SP53 and SK1-KD MCL cell lines. (**A**) Lipidomic analysis of lipid classes in SP53, SK-KD1, and SK1-KD2 cell lines. Three biological replicates of each cell line were analyzed using differential mobility spectroscopy to determine the total number of moles of each lipid class present per billion cells. The average number of moles per billion cells for cardiolipins of different (**B**) acyl chain lengths, (**C**) saturation, and (**D**) select species were graphed for each of the three cell lines. Data was analyzed by one-way ANOVA * *p* < 0.05; ** *p* < 0.001; *** *p* < 0.0001.

NKT cells can be activated or inhibited by glycolipids presented in the context of CD1d. The lipids can be microbial in origin or they can be self-lipids. To examine differences between SP53, SK1-KD1, and SK1-KD2, we analyzed their lipid profiles by mass spectrometry. We found increases in several different lipid classes in the SK1-KD1 and SK1-KD2 cell lines compared to SP53, the most significant of which was cardiolipin (Figure 5A). Cardiolipin is a tetra-acylated phospholipid found in both bacteria and the inner-mitochondrial membrane. Therefore, we postulated that cardiolipin has the potential to become an activating lipid for NKT cells and its increase in SK1-KD1 and SK1-KD2 could play a role in the increased NKT cell responses previously observed.

Next, we sought to further characterize the differences in cardiolipin by examining characteristics such as acyl chain length and saturation. We found that the differences between the SK knockdown cell lines and SP53 were greatest for cardiolipins with 16 and 18 carbon acyl chains (Figure 5B). In addition, SK knockdown cells had increases in saturated, mono-unsaturated, and poly-unsaturated cardiolipins (Figure 5C). Finally, we identified several subspecies of cardiolipin that showed significant differences in expression levels (Figure 5D).

### 3.5. Cardiolipin Stimulates Type 1 NKT Cells

**Figure 6:**
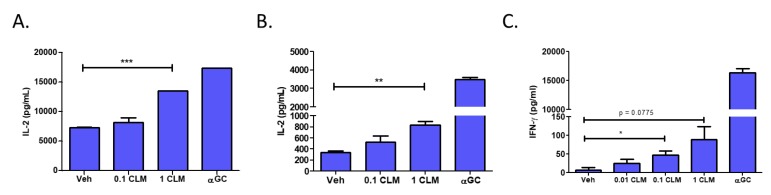
Treatment with cardiolipin enhances NKT activation. L-CD1d cells were pulsed with a mixture of cardiolipin species (CLM) for 4 h at 37 °C prior to co-culture with (**A**) DN32.D3 or (**B**) N38-3C3 for 24 h. IL-2 production was measured by ELISA. (**C**) Mouse splenocytes were pulsed with CLM for 4 h at 37 °C prior to co-culture with mouse liver mononuclear cells (MNCs) for 48 h. IFN-γ levels in the supernatant was then measured by ELISA. Each ELISA was conducted in triplicate. Data was analyzed using a student’s *t*-test. * *p* < 0.05; ** *p* < 0.005; *** *p* < 0.0001.

To test whether cardiolipin can serve as an activating ligand for type 1 NKT cells, we examined the ability of a mixture of bovine heart derived-cardiolipin to stimulate NKT cells. We pulsed L-CD1d cells with two concentrations of the cardiolipin mixture for four hours prior to co-culture with NKT cell hybridomas. We found that increased levels of cardiolipin resulted in increased IL-2 production by both DN32.D3 (Figure 6A) and N38-3C3 (Figure 6B) NKT cell hybridomas. Next, we tested the ability of cardiolipin to activate NKT cells using a primary cell culture system. Splenocytes from C57BL/6J mice were pulsed with cardiolipin for four hours. The majority of T cells in the liver are NKT cells, therefore, we co-cultured the cardiolipin loaded splenocytes with liver mononuclear cells and assessed IFN-γ production. Importantly, we found that increased levels of cardiolipin resulted in increased cytokine production (Figure 6C). Taken together, this data suggests that cardiolipin may be an activating lipid for NKT cells.

**Figure 7:**
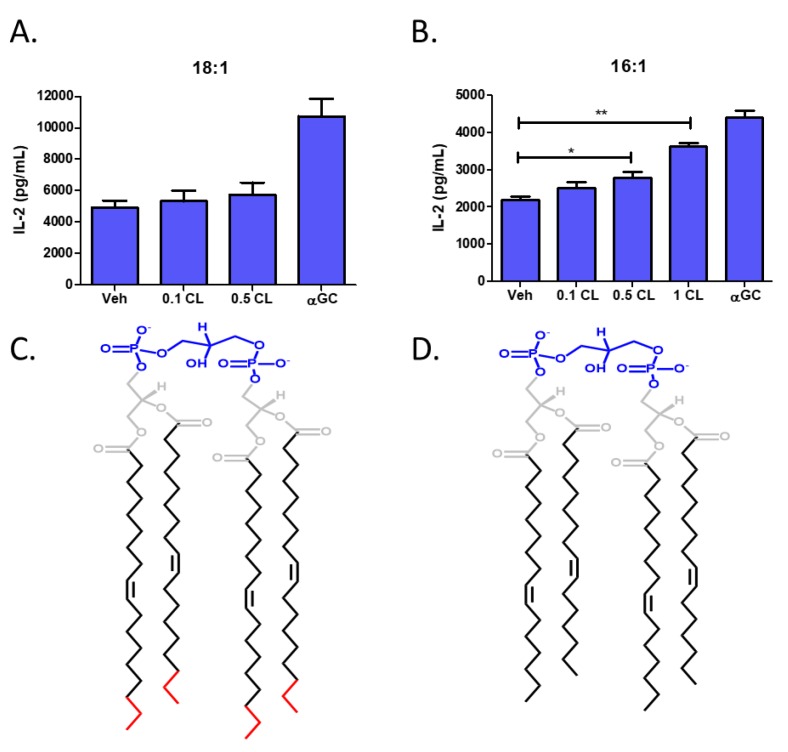
Identification of cardiolipin species that activates type 1 NKT cells. L-CD1d cells were pulsed in the presence of either purified (**A**) 18:1 or (**B**) 16:1 cardiolipin for 4 h at 37 °C prior to co-culture with DN32.D3 for 24 h. IL-2 production was then measured by ELISA. Each ELISA was conducted in triplicate. Structures of 18:1 and 16:1 cardiolipin are shown in (**C**), (**D**) respectively with the head group in blue, the acyl chain in black, and the extra carbons in red. Data was analyzed using a student’s *t*-test. * *p* < 0.05; ** *p* < 0.005.

To further delineate the species-specific differences observed in our lipidomic analysis, we utilized two purified cardiolipin species, 18:1 and 16:1, that we predicted may be responsible for NKT cell activation. We pulsed L-CD1d cells with increasing concentrations of either 18:1 or 16:1 cardiolipin for 4 h prior to co-culture with DN32.D3 NKT cell hybridomas. There were no significant changes in NKT cell responses following treatment of antigen presenting cells with 18:1 cardiolipin (Figure 7A). However, NKT cell responses were significantly increased in the presence of 16:1 cardiolipin (Figure 7B). The two cardiolipin species are identical aside from an extra two carbons on the acyl chain of 18:1 cardiolipin (Figure 7C) relative to 16:1 cardiolipin (Figure 7D). These data support the notion that specific species of cardiolipin may induce NKT cell responses while other species may not be stimulatory. Further studies are needed to elucidate all the structures that are activating compared to those that do not result in NKT cell activation.

## 4. Discussion

In this study, we present evidence demonstrating that increased S1P signaling results in changes in B cell antigen processing and presentation leading to reduced NKT cell activation. We also showed that blocking either S1P production or signaling permits NKT cell-mediated killing of MCL. Next, we showed that blocking S1P production through the knockdown of SK resulted in changes in several different lipid species, including an increased level of cardiolipin. Finally, we identified cardiolipin, specifically 16:1 cardiolipin, as a novel NKT agonist. These data further suggest that removal of tumor associated immunosuppressive factors, such as S1P, may be efficacious as a targeted therapeutic, by restoring presentation of endogenous NKT cell agonists.

S1P was identified as a lipid metabolite that induces an increase in intracellular calcium and acts through inside out signaling [29,30]. S1P is generated from sphingolipids, essential serum membrane lipids which are concentrated in lipid rafts. It is synthesized via sphingomyelinase conversion of sphingomyelin into ceramide, then ceramidase converts ceramide into sphingosine and lastly sphingosine kinase converts sphingosine into S1P. S1P is involved in malignant transformation, cancer cell proliferation, inflammation, vasculogenesis and resistance to apoptosis. Over-expression of SK has been reported to induce malignant transformation, promote tumor proliferation, and be associated with poor outcomes in multiple tumor types [31,32,33,34,35,36,37,38]. Importantly, we have found that both SK1 and SK2 are highly expressed in lymphoma cell lines and that inhibiting SK1 restores NKT cell mediated killing and cytokine responses to MCL. Another group has found that inhibiting SK1 results in apoptosis of MCF7, a breast cancer cell line [45]. In addition, an important role for the S1P pathway as a carcinogenic marker in a colon carcinogenesis model in rats has been shown, due to the up-regulation of Cox-2 and PGE2 [35].

Several studies have recently evaluated S1P as a therapeutic target for the treatment of lymphoid malignancies. It was shown that blocking S1P signaling with an agonist, FTY-720, resulted in increased apoptosis in multiple myeloma cells [46], induced cell death in chronic lymphocytic leukemia [47], and inhibited MCL pathogenesis in a SCID mouse model [40]. In addition, it was shown that S1P inhibited NK cell-mediated lysis of the human melanoma cell line Hs294T [48], the Burkitt’s lymphoma cell line Raji, and the myeloid leukemia cell line K562 [49]. This effect of S1P was reversed by FTY720 and a second S1P_1_ antagonist, SEW2871.

Our data suggest that inhibiting S1P signaling may be a beneficial therapeutic modality in the treatment of MCL because the blockade of S1P signaling could both inhibit proliferation of MCL and restore NKT cell mediated cytotoxic responses to MCL. We found that pretreatment with SEW2871 and W146 had minimal effects on CD1d cell surface expression (data not shown), but restored NKT cell-mediated killing of MCL. S1P is cleaved irreversibly to hexadecenal and phosphoethanolamine (PE), by S1P lyase. PE can bind to CD1d molecules [50,51]. A recent study has shown that intracellular pools of S1P are localized in the Golgi, as well as in late endosomal compartments [44]. We and others previously demonstrated that CD1d molecules must traffic through endosomal compartments in order to process and present antigen to NKT cells [41,52,53]. We also previously reported that the activation of MAPK signaling pathways can alter CD1d-mediated NKT cell activation [54]. Perhaps high intracellular pools of S1P in endosomal compartments of MCL alter CD1d-mediated antigen processing and presentation.

Aberrant S1P signal transduction and intracellular transit in B cell lymphomas may potentially result in the deregulation of CD1d-mediated activation NKT cells by lymphomas, contributing to the NKT-cell activation deficit described here. Several possible mechanisms could contribute to the failure of MCL cells to induce cytotoxic NKT cell responses, including alterations in lipid ligands, modifications in intracellular trafficking of CD1d, and upregulation of MAPK or BCL-2 pro-survival signaling in MCL cells. Of these possible mechanisms, our data demonstrates that alterations in lipid ligands plays a role. Specifically, we found that several lipid classes, including cardiolipin, phosphatidylcholine, phosphatidylethanolamine, and sphingomyelin were all upregulated after knockdown of SK in an MCL cell line. Importantly, reducing SK1 resulted in the presentation of an endogenous activating antigen (Figure 4A), without altering CD1d cell surface expression (Figure 4C). Thus, these data suggest alterations in the repertoire of lipid classes can lead to the presentation of an endogenous activating NKT cell ligand.

Cardiolipin is a tetra-acylated phospholipid that can be of either prokaryotic or eukaryotic origin and regulates SK activity [55]. In prokaryotes, cardiolipin is a component of bacteria, specifically found in the cell wall of gram-negatives. In eukaryotes, cardiolipin is only found in the inner mitochondrial membrane [56]. Cardiolipin is known to bind to human CD1d molecules [57]. Cardiolipin has been found to stimulate CD1d-restricted γδ T cells in mice [56]. In addition, bacterial-derived cardiolipin was found to possibly stimulate type II NKT cells, which are CD1d-restricted but do not express the semi-invariant NKT TCR associated with type I NKT cells [58]. An early study reported that cardiolipin was not able to stimulate human type I NKT cells [59]; however, this study used a synthetic CD1d-Fc fusion system to present the different lipid classes which may not completely recapitulate antigen processing and presentation in live cells. In our study, we found that pulsing CD1d-expressing cells with cardiolipin induced NKT cell activation. In addition, we found that certain cardiolipin species were able to specifically activate type I NKT cells, in contrast to other species (Figure 6A,B). This was intriguing as the acyl chain structure is known to change the ability of phospholipids to bind CD1d and stimulate NKT cells [51], which also may explain why previous studies using bulk mixtures of cardiolipin species did not identify it as an activating ligand. Moreover, changing the saturation status of the cardiolipin chain causes cardiolipin to flip between activating and inhibiting TLR4 signaling [60]. In good agreement with these studies demonstrating the importance of cardiolipin’s acyl chain structure in its function, we also have identified a unique cardiolipin species (16:1) that is able to serve as an activating ligand for type 1 NKT cells.

## 5. Conclusions

In summary, S1P plays a role in many pathways involved in tumor development and progression. Our data suggest that S1P receptor signaling inhibitors can be used to both inhibit lymphoma growth and to restore the anti-tumor functions of primary human NKT cells. Our studies also identified a novel mechanism by which S1P receptor signaling inhibits CD1d-mediated NKT cell activation. Future studies should examine the in vivo effects of SK knockdown to determine if they mirror the in vitro systems. In addition, the mechanisms underpinning cardiolipin acyl chain regulation in MCL along with its relationship to sphingosine kinase and S1P signaling needs to be further elucidated. These studies will define the potential uses of S1P signaling modulators and enhance clinical approaches for NKT cell-based immunotherapy for the treatment of MCL.
